# Inhibition of Return Biases Orienting During the Search of Complex Scenes

**DOI:** 10.1100/tsw.2003.03

**Published:** 2003-03-17

**Authors:** W. Joseph Macinnes, Raymond M. Klein

**Affiliations:** Faculty of Computer Science, Dalhousie University, Halifax, Nova Scotia, Canada

**Keywords:** inhibition of return, attention, visual search, oculomotor preparation

## Abstract

In Klein and MacInnes[1], observers searched a complex scene for a camouflaged target. Reflecting Inhibition of Return (IOR) observers were slower to detect and saccade to uncamouflaged probes that interrupted active search when these were placed in the vicinity of a recent fixation. To explore the generality of this finding of IOR during search, we changed the mental state of the observer at the time of the probes by instructing observers to inspect the scene until they found something interesting and stop there. After this voluntary cessation of search, we presented an uncamouflaged probe that observers were required to foveate. Extending our previous demonstration, we observed a relative increase in the time required to locate these probes when they were in the general region of a previous fixation so long as the scene was maintained. When the scene was removed, probe reaction time was unaffected by distance from the last fixation. The pattern of results supports the proposal that IOR biases overt orienting during search.

## INTRODUCTION

Following visual orienting toward a location in space there is a delay in responding to targets at that location, so long as attention is withdrawn from the previously cued location[2]. This inhibitory aftereffect of visual orienting has come to be called *inhibition of return* (IOR)[3,4]. Klein[5] reasoned that if IOR biases orienting toward new locations in the environment, it could serve to facilitate visual foraging behavior - search. Although there were challenges to this foraging proposal[6,7,8], these challenges have been rebutted[9,10,11,12,13,14] and direct evidence for the proposal has been provided by Klein and MacInnes[1] who explored IOR using

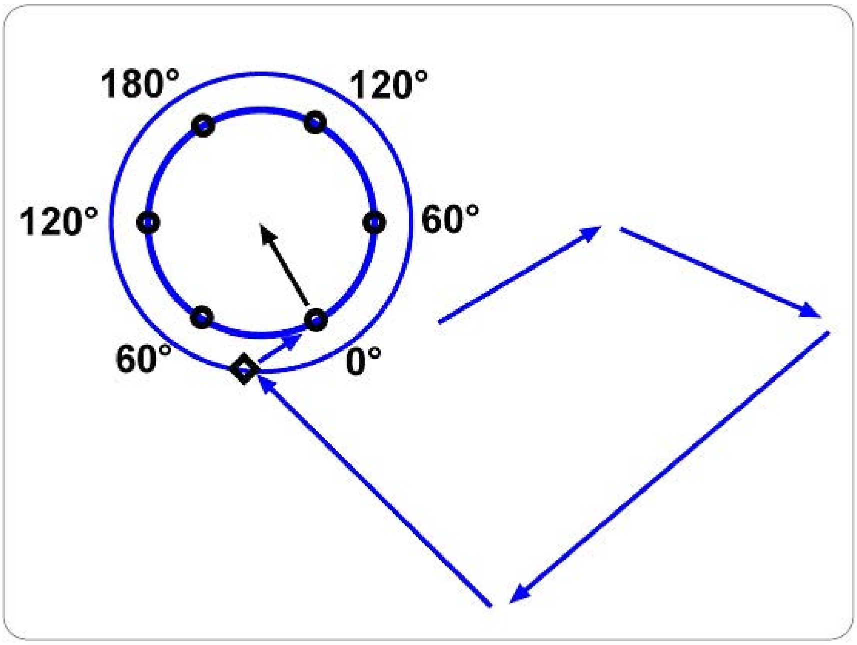

**FIGURE 1**. Following a sequence of search saccades (shown as arrows) one black annulus probe stimulus was displayed at any one of 6 equally spaced locations centered on the current fixation (shown at the terminus of the final black arrow). In the one-back condition, illustrated here, the immediately preceding fixation was one of the 6 randomly selected probe locations (along the inner blue circle), which are labeled according to their angular distance from the one-back fixation. In the two-back condition, the probes would have been placed along the outer blue circle with the 0 degree probe at the two-back fixation (shown by the diamond). Klein & MacInnes [1] used both the one- and two -back conditions. Only the one back condition was used here.

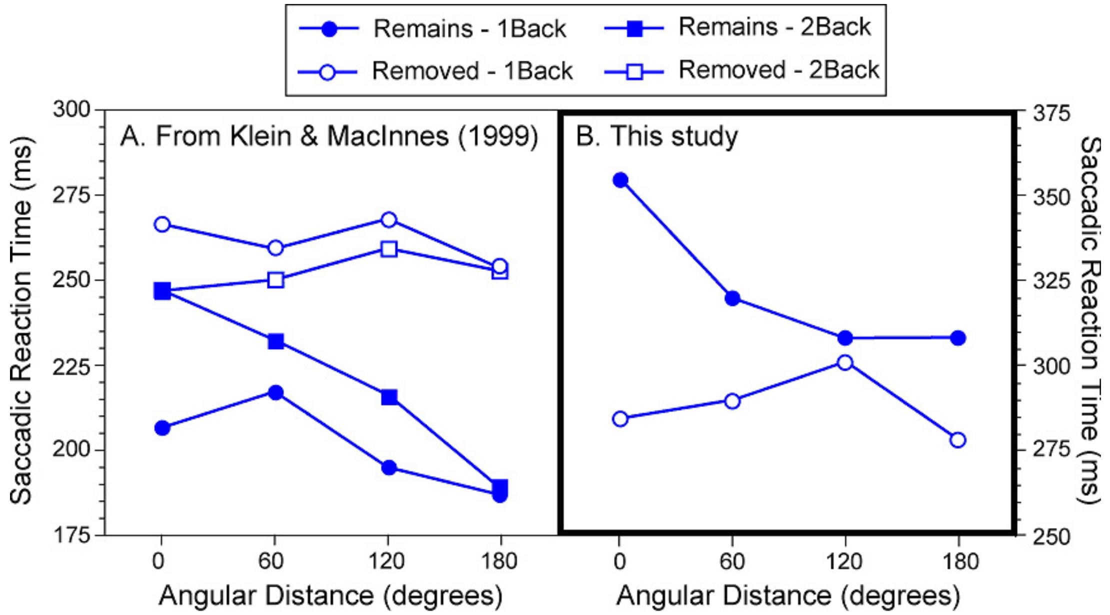

**FIGURE 2.** Saccadic Reaction time to foveate the probe when it was acquired in a single saccade from Klein and MacInnes ([1], Experiments 1 & 2 (a) and the current study (b). In this and all subsequent data figures, the data from the present study are highlighted by the thicker outline box.

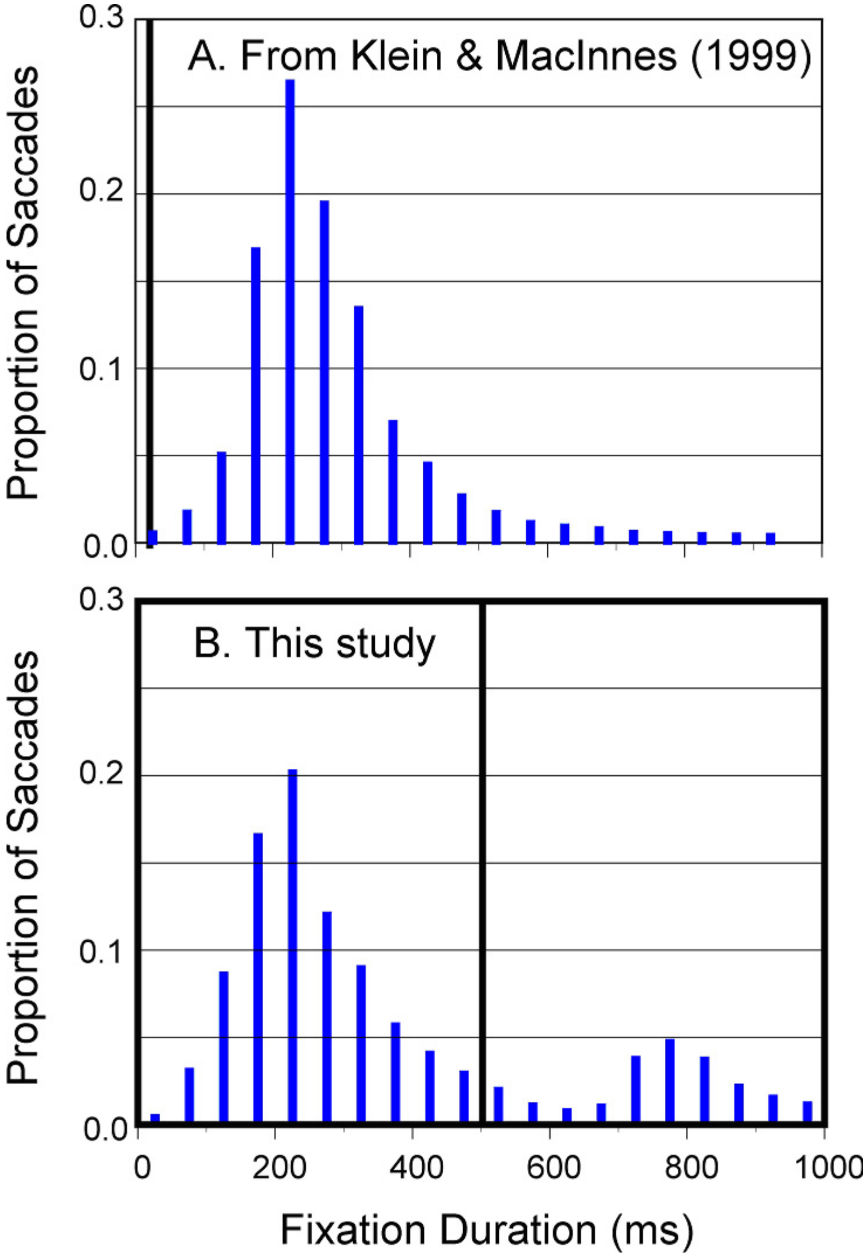

**FIGURE 3**. Proportion of saccades leading to different fixation durations is plotted as histograms. (a) Klein and MacInnes [1], experiments 1 and 2 combined; (b) current study. The time of probe delivery is shown by black vertical lines positioned at 20 ms and 500 ms, in (a) and (b), respectively.

overt orienting during search for a camouflaged target in a complex visual scene (Waldo or the Wizard from the Where's Waldo™ series of books). After several freely made search saccades, the original fixation stimulus reappeared unpredictably while the scene was removed or maintained and participants were required to detect and foveate this probe (see Fig. 1). When the probe was acquired in a single saccade following its presentation, reaction time (RT) was slower for probes in the general region of a previous fixation (see Fig. 2A, filled symbols). This dependence of probe RT on the relationship between the probe's location and a previous fixation was eliminated however, if the search array was removed when the probe was presented (see Fig. 2A, open symbols). In agreement with recent studies showing that IOR following visual search

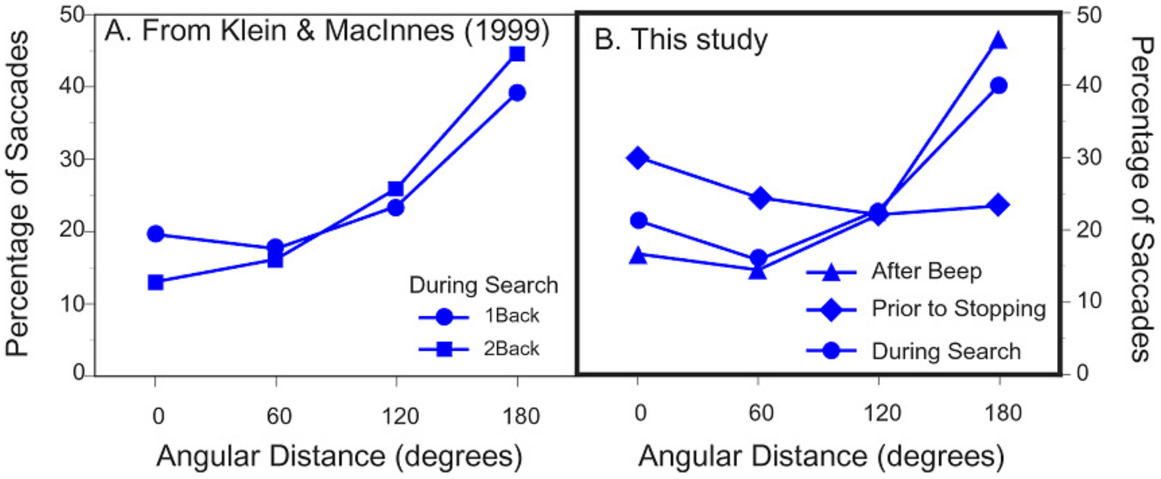

**FIGURE 4**. Percentage of pre-probe saccades executed in various directions based on the angular distance from a previous fixation: (a) from Klein and MacInnes ([1], Experiments 1 and 2), (b) the current study. In the current study: 'after beep' refers to saccades immediately after a voluntary cessation of search (signaled by a 500 ms fixation duration) and signal to resume searching; 'prior to stopping' refers to the saccade immediately before such a cessation; and 'search' refers to all other pre-probe saccades.

depends upon maintenance of the search array[10,12] this pattern confirms Tipper et al.'s[14] suggestion that it should because IOR is coded in object or scene coordinates[15,16].

In the present experiment we sought to replicate this finding while significantly altering the mental state of the observer at the time the probes were presented. This was accomplished by changing the instructions, timing, and conditions for probe delivery, while maintaining the paradigm of free search of a complex scene. In the previous study, subjects searched complex scenes for a specific target (Waldo or the wizard) and the probes were delivered without warning 20 ms after a pseudo-randomly selected fixation had begun. Processing of the probe thus took place in parallel with oculomotor programming of the next search saccade (see Fig. 3A). In the present study, participants were instructed to search each scene for 'something interesting' and to stop there. Rather than interrupting search, probes were delivered when the observer had stopped overtly searching and, therefore, when it was unlikely that the observer would be actively initiating his or her next saccade. This timing of the probes and nature of the observer's task will allow the results from this study to shed light on two issues that were raised in the earlier study.

In Klein and MacInnes[1] probe reaction time was faster when the scene remained present than when it was removed. This was surprising because probes would have been more conspicuous in an empty field than superimposed on the Where's Waldo$^{TM}$ picture. Although it was noted that this finding might be explained in terms of a sensory effect ("the global transient associated with removal of the scene might have masked the local transient associated with the onset of the probe"), the following motoric hypothesis was preferred: "removing the visual data used to compute oculomotor parameters for the Waldo search might have caused a refractory period for the programming of subsequent saccades, until clearing and resetting of the visual-motor representations was completed." With the present instructions, observers would be unlikely to be initiating a scene-related saccade at the time of the probe because probes are delivered after the observer had stopped searching, yet the probe's onset could still be masked by the simultaneous global transient from scene removal. Hence, if the motoric hypothesis is correct, the disadvantage for the scene-removed condition should be eliminated. Reingold and Stampe[17] recently reported that a visual change simultaneous with the onset of a target delays voluntary and reflexive saccade latencies by about 20 ms, and proposed that this delay is caused by superior colliculus-mediated saccadic inhibition. Similar to the prediction from the sensory/masking proposal, if such saccadic inhibition due to the full-field transient associated with scene-removal were responsible for the disadvantage Klein and MacInnes[1] found for scene removal, then this disadvantage will be replicated in the present experiment.

Klein and MacInnes[1] reported that saccades made by participants while they freely inspected each scene (preprobe saccades) showed a strong directional bias (see Fig. 4A) away from previously fixated regions. This raises an interesting *chicken-and-egg* puzzle. On the one hand, the inhibitory tags left at each fixated location when gaze shifts to a new one might serve to bias subsequent search behavior away from these previously fixated locations. Alternatively, during search of a complex scene, a natural tendency to saccade away from previously studied regions may result in delayed detection and localization of targets presented in previously fixated regions. To the degree that the oculomotor system uses a winner-take-all algorithm mediated by lateral inhibition, a preparatory bias in one direction would result in inhibition of orienting in the opposite direction and vice versa. Simply put, a question that remains is whether IOR causes the biased oculomotor programming or the biased oculomotor programming causes IOR. In the present study, rather than probing at the start of a fixation in the middle of a sequence of search-saccades, probes were not shown until observers had signaled they had stopped searching and fixated something interesting. By greatly reducing, if not eliminating, the active programing of the next search-saccade at the time of the probe delivery, the present design addresses the chicken-and-egg puzzle. If biased oculomotor preparation causes IOR, then IOR should be reduced in this experiment compared to that seen in Klein and MacInnes[1], if not completely eliminated. In contrast, if IOR causes search to be biased away from previous inspections, then a robust pattern of IOR should be obtained.

## METHODS

### Participants

Twelve adult volunteers participated in the experiment; all were students of Dalhousie University and participated for class credit.

### Stimuli and Apparatus

Stimuli were the eight scenes (scanned from three Where's Waldo™ books[1] and modified for our requirements) that were used by Klein and MacInnes[1]. The fixation and probe stimuli were black circles subtending 0.5º of visual angle with 0.1º diameter transparent center. Stimuli were presented on a 17", SVGA, ViewSonic monitor (640 × 480 pixels, 256 colors) in an area 25.0º (width) × 19.4º (height) at a viewing distance of 71 cm. Scenes were drawn or modified in a single screen refresh.

An EyeLink™, video-based, eye-tracking system was used to sample each participant's gaze position every 4 msec with a resolution of 0.1º or better. Using this system, information about changes in gaze direction was available to the experimental program within approximately 20 ms.

### Design and Procedure

The experiment consisted of a block of 288 trials in which each of the eight Waldo pictures was used equally often. Each trial began when the subject pressed the space bar to indicate that they were looking at the central fixation circle. If fixation was stable, a picture was displayed. The fixation stimulus remained present for a further 700 ms and participants were instructed to maintain fixation until it disappeared. Any saccades or drifts (of more than 1º) that occurred during this period resulted in a warning beep and the trial was recycled. Otherwise, participants were free to search the scene until they found something 'interesting', at which time they were to remain fixated on the interesting item. On most trials, after we thought participants had stopped, one of two things happened: a short beep occurred, indicating that participants were to resume searching for something else interesting, or the fixation disk (probe) would reappear. Participants were instructed to foveate this disk as quickly as possible whenever they detected it.

Whenever a fixation duration exceeded 500 ms, it was assumed the observer had *stopped*. This duration was selected based on the distribution of fixation durations observed in our previous study where finding Waldo was the cover story for search (see Fig. 3A). At this point, the probe was presented or the *continue* beep was sounded. On half of probe trials, the search picture remained visible and the probe was superimposed on the picture; on the remaining trials, the picture was removed, and the probe was presented singly in a uniform gray field. The probe, when presented, appeared at a location determined by the current and immediately prior (one-back)[2] fixation positions, as illustrated in Fig. 1. The current gaze position became the center of an imaginary circle with a radius equal to the distance between the current and previous fixation. Possible probe locations on the circumference of this circle included the previous fixation location (0º) as well as five other locations, equally spaced around the circumference of the circle (60, 120, 180, 240 and 300º, based on their angular distance from the one-back fixation). All six locations were equally likely to contain a probe. If the calculated radius of this imaginary circle was less than 1º or would place any one of the possible probes off the screen, or if fewer than 2 saccades had been made since the beginning of the trial, probe presentation was delayed and the continue beep signaled the subject to find something else to look at. If no suitable gaze position could be found within 15 saccades, the trial was terminated.

A saccade was said to acquire the probe if the landing coordinates of the saccade were within 1º of the probe's center. All other fixations were classified as misses. The primary dependent variable was the saccadic reaction time (SRT) to acquire the probe when it was acquired within one saccade. Outliers were removed from the data using a modified recursive procedure[18].

## RESULTS

### Probe Presentation

As noted above, probes were not delivered until a fixation exceeded 500 ms (and certain other conditions were satisfied, see methods). To confirm that the 500-ms criterion was adequate for identifying trials on which the observer’s eye movements had come to a stop, we examined the distribution of fixation durations (Fig. 3B). Keeping in mind that all fixations over 500 ms resulted in the delivery of a probe or a signal to continue, the bimodal distribution clearly shows that the majority of free search fixations had terminated before the probe presentation or continue signal at 500 ms.

### Probe Acquisition Performance

Trials were excluded from the analysis of probe RT if there was an eye blink (19.9%) or if a probe could not be delivered (15.6%). Considering the remaining trials, probes were acquired in a single saccade slightly more often when the scene was removed (83.6%) than when it remained present (77.6%) ($F[1,11] = 3.8$, $p < 0.08$)[3].

Mean saccadic reaction time (SRT) for trials on which the probe was acquired in the first saccade is shown in Fig. 2B. The data were subjected to a 2 (scene removal) × 4 (angular distance[4] from the previous fixation) repeated measures analysis of variance (ANOVA). There was a significant interaction between scene removal and angular distance ($F[3,33] = 3.21$, $p < 0.04$), reflecting differences in reaction time as a function of distance when the scene remained ($F[3,33] = 2.95$, $p < 0.05$), but not when it was removed ($F[3,33] = 1.774$, $p > 0.15$). The results are consistent with previous findings suggesting that IOR is coded in scene or object, rather than retinal, coordinates. When the scene remained present, RT was slowest for targets presented at the location of the previous fixation and decreased monotonically as the angular distance from this location increased. Comparing the end points (0 and 180º), which correspond most closely with the cued and uncued locations in a typical IOR study, there was a cost of 47 ms for returning to the most recently fixated ($p < 0.004$) location.

## Preprobe Saccades

All saccades prior to the probe were sorted into six 60º bins based on the angular difference between the vectors of the current saccade and the preceding one. The 0º bin was centered on the vector back toward the previous fixation; the 180º bin was centered on the vector continuing in the same direction. These data were further divided into saccades that led to stopping (a fixation duration > 500 ms); saccades that resumed search after stopping (when we signaled the subject to continue); and all other saccades, which are called search saccades. The data from the present study are shown in Fig. 4B along with corresponding data from the previous study (Fig. 4A). As before there was a clear directional bias away from the immediately preceding fixation for search saccades ($F[3,132] = 75.5$, $p > 0.0001$) and for saccades that resumed search. In contrast, the saccades that led to cessation of search were relatively unbiased, and to the extent that there is a bias for these saccades there is a slight, but significant ($F[3,33] = 4.221$, $p < 0.02$) trend back toward the previous fixation[5].

## DISCUSSION

The two unresolved questions from Klein and MacInnes[1], raised in the introduction, can be addressed by these findings. (1) Why was probe RT slower when the scene was removed? (2) Does IOR bias oculomotor preparation or vice versa? We will begin with a third question, i.e., why was IOR less robust in their one-back than two-back conditions (see Fig. 2A)?

Measured as the difference between the 0 and 180º conditions, Klein and MacInnes[1] found less robust IOR at the most recently fixated location (one-back; 20 ms) than at the location prior to that one (two-back; 57 ms). This difference may have been related to the greater passage of time since the 0º probed location had been attended in the two-back case (about 330 ms for two-back vs. 50 ms for one-back). Alternatively, the critical variable might have been the ordinal relation between fixations and the probe. The present study maintained the ordinal position of the previous one-back condition while increasing the time between the critical fixation and the probe (to about 530 ms). The robust IOR we obtained with these conditions (47 ms) strongly implicates the passage of time as the critical variable. Although an explanation is not entailed by this pattern, one might be found in the biphasic pattern, often seen in cuing studies, of facilitation immediately after a cue followed by a long lasting inhibition (e.g., see Fig. 1 from [19] and Fig. 3 from [4]). Perhaps there was still some facilitation in the region of the previous fixation when probes were delivered there within 50 ms of the departing saccade.

The task and timing of probe delivery were designed to minimize the likelihood that participants would be actively initiating a saccade when the probes were presented. Several findings from this study converge on the conclusion that this was accomplished.

The main effect of scene removal, although not significant ($F[1,33] = 2.39$, $p < 0.15$), represents an important departure from our previous study. Whereas Klein and MacInnes[1] found a significant RT cost (47.8 ms) associated with removing the scene, here we find a nonsignificant advantage of 34.4 ms. This difference was confirmed using an ANOVA with study as a between-subject factor which revealed there was a highly significant interaction between scene removal and study ($F[1,24] = 13.05$, $p < 0.002$). This difference is consistent with our earlier proposal that the cost was due to the need to clear and reset the visual-motor machinery when the data from the visual array that were serving as the basis for the upcoming search saccade had been unpredictably removed. That this disadvantage was eliminated when observers stopped searching strongly supports the notion that at the time of the probes in the present study, the information in the visual array was not being actively used to compute saccade metrics. Since the participants in the present study stopped searching of their own volition, there would be less likelihood of an about-to-be-initiated saccade to clear. This difference, combined with the fact that saccades in the scene removed condition did not show inhibition, strongly suggests that subjects were not about to initiate a saccade at the time of probe presentation[6].

It is also worth noting that RTs were, overall, slower in this experiment (mean = 305.9) than in our previous search study (mean = 236.2). This difference is likely due to the high level of oculomotor engagement upon fixation[20] when subjects have stopped searching and are maintaining fixation (as in the present study) as compared to a state of oculomotor preparation and disengagement from fixation when probes are delivered in mid-search (as in the previous study).

Finally, in order to determine if saccades were influenced by an orderly search similar to that in reading, all free search (pretarget) saccades were plotted according to their absolute direction on the screen and grouped into eight bins. The percentages of saccades falling into each bin (see Fig. 5A,B for data from Klein and MacInnes[1] and 5C for data from this study.) do show a horizontal bias, but it fits nicely with the rectangular screen (640 × 480) used for presentation. As a point of comparison, the results were compared to a reading study[21] conducted using the same equipment and display settings. Although this does not rule out all strategies, it shows that subjects were definitely not using a strategy as formalized as that in reading (see Fig. 5D). If a strategy to scan the array horizontally and/or vertically was causing our observers to manifest an IOR-like pattern when probes were delivered in previously fixated regions, then the degree to which this strategy was apparent and the magnitude of IOR should be positively correlated. To further explore this possibility we calculated a bias score for each subject. Preprobe saccades were sorted according to whether their absolute direction had a primarily horizontal or vertical as opposed to oblique vector, and the excess of horizontal plus vertical saccades over obliques ones was expressed as a percentage. The correlation between this score and IOR (near locations, 0 and 60°, minus far locations, 120 and 180°) in the scene remains condition was –0.29. Although not significant, the trend suggests that the more deliberately subjects searched for an interesting object, the less IOR they showed.

On the basis of the change in task and the analyses described above, it seems warranted to assume that the probes in this study were presented while the participant was in a completely different state of motor preparation from that in our previous experiment. Because IOR was robust in this study while active oculomotor preparation was minimized, it is more likely that IOR causes the oculomotor bias during free search (Fig. 4) than that IOR is caused by this bias[7].

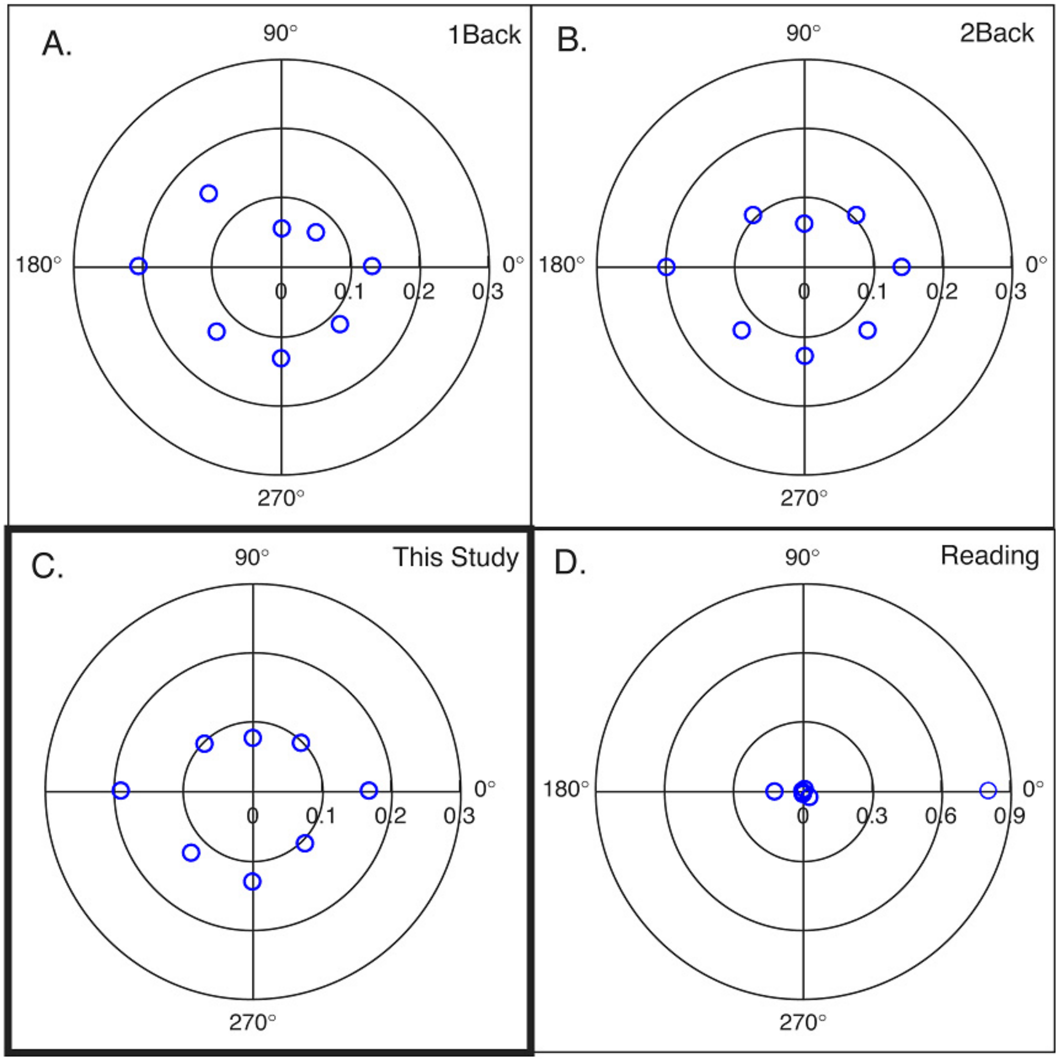

**FIGURE 5**. Percentage of saccades executed in various *absolute* directions. (a) search pattern from Klein and MacInnes ([1], Experiment 1); (b) search pattern from Klein & MacInnes ([1], Experiment 2); (c) search pattern from the current study; (d) search during reading (from [20]). Note the scale difference in (d).

Converging evidence for this conclusion is provided by Peterson et al.[22,23]. Single saccade targets were presented one at a time, until a critical point when two targets were presented. One of these was displayed in a new location whereas the other was presented in a location that had earlier been occupied by a target. IOR was observed as a higher probability that saccades in response to this critical dual-target would be directed away from the previously fixated location. In this paradigm, the scan path is controlled by the experimenter and, since participants were not aware of where the next target would be presented, active preparation was unwarranted. Thus, the IOR obtained by Peterson et al. was, like that obtained here, unlikely to have been caused by differential oculomotor preparation.

## ACKNOWLEDGEMENTS

The research reported here was supported by grants to R. Klein from the Natural Sciences and Engineering Research Council (NSERC) of Canada and from the McDonnell-Pew Program in Cognitive Neuroscience. W.J. MacInnes was supported by NSERC and Killam Scholarships.

## References

1. Klein, R.M. and MacInnes, W.J. (1999) Inhibition of return is a foraging facilitator in visual search. *Psychol. Sci*. **10(4)**, 346–352.
2. Posner, M.I. and Cohen, Y. (1984) Components of visual orienting. In *Attention and Performance X*. Bouwhuis, D., Ed. Erlbaum, London. pp. 531–556.
3. Taylor, T.L. and Klein, R.M. (1998) On the causes and effects of inhibition of return. *Psychon. Bull. Rev.* **5(4),** 625–643.
4. Klein, R.M. (2000) Inhibition of return. *Trends Cogn. Sci*. **4(4)**, 138–147.
5. Klein, R.M. (1988) Inhibitory tagging system facilitates visual search. *Nature* **334(6181)**, 430–431.
6. Wolfe, J.M. and Pokorny, C.W. (1990) Inhibitory tagging in visual search - a failure to replicate. *Percept. Psychophys*. **48(4)**, 357–362.
7. Klein, R.M. and Taylor, T.L. (1994) Categories of cognitive inhibition with reference to attention. In *Inhibitory Processes in Attention, Memory, and Language.* Dagenbach, D. and Carr, T.H., Eds. Academic Press, San Diego, CA. pp. 113–150.
8. Pratt, J. and Abrams, R.A. (1995) Inhibition of return to successively cued spatial locations. *J. Exp. Psychol. Hum. Percept. Perform*. **21(6)**, 1343–1353.
9. Lupianez, J., Milan, E.G., Tornay, F.J., Madrid, E., and Tudela, P. (1997) Does IOR occur in discrimination tasks? Yes, it does, but later. *Percept. Psychophys*. **59(8)**, 1241–1254.
10. Müller, H.J. and von Mühlenen, A. (2000) Probing distracter inhibition in visual search: inhibition of return. *J. Exp. Psychol. Hum. Percept. Perform*. **26(5)**, 1591–1605.
11. Snyder, J.J. and Kingstone, A. (2000), Inhibition of return and visual search: how many separate loci are inhibited? *Percept. Psychophys*. **62(3)**, 452–458.
12. Takeda, Y. and Yagi, A. (2000) Inhibitory tagging in visual search can be found if search stimuli remain visible. *Percept. Psychophys*. **62(5)**, 927–934.
13. Tipper, S.P., Weaver, B., and Watson, F.L. (1996) Inhibition of return to successively cued spatial locations: commentary on Pratt and Abrams (1995*). J. Exp. Psychol. Hum. Percept. Perform*. **22(5)**, 1289–1293.
14. Tipper, S.P., Weaver, B., Jerreat, L.M., and Burak, A.L. (1994) Object-based and environment-based inhibition of return of visual-attention. *J. Exp. Psychol. Hum. Percept. Perform*. **20(3)**, 478–499.
15. Abrams, R.A. and Dobkin, R.S. (1994) Inhibition of return: effects of attentional cueing on eye- movement latencies. *J. Exp. Psychol. Hum. Percept. Perform*. **20(3)**, 467–477.
16. Tipper, S.P., Driver, J., and Weaver, B. (1991) Object-centered inhibition of return of visual attention. *Q. J. Exp. Psychol. A* **43(2)**, 289–298.
17. Reingold, E.M. and Stampe, D.M. (2002) Saccadic inhibition in voluntary and reflexive saccades. *J. Cogn. Neurosci*. **14(3),** 371–388.
18. van Selst, M. and Jolicoeur, P. (1994) A solution to the effect of sample-size on outlier elimination. *Q. J. Exp. Psychol. A* **47(3)**, 631–650.
19. Samuel, A.g. and Kat, D. (in press) Inhibition of return: A graphical meta-analysis of its timecourse, and an empirical test of its temporal and spatial properties. Psychonomic. Bull. & Rev.
20. Taylor, T.L., Kingstone, A., and Klein, R.M. (1998) The disappearance of foveal and nonfoveal stimuli: decomposing the gap effect. *Can. J. Exp. Psychol.* **52(4)**, 192–200.
21. Saint-Aubin, J. and Klein, R.M. (2001) Influence of parafoveal processing on the missing-letter effect. *J. Exp. Psychol. Hum. Percept. Perform*. **27(2)**, 318–334.
22. Peterson, M.S., McCarley, J.S., Kramer, A.F., Irwin, D.E., and Wang, R.F. (2000) Visual search has memory. In Annual Meeting of the Psychonomics Society, New Orleans.
23. Peterson, M.S., McCarley, J.S., Kramer, A.F., Irwin, D.E., and Wang, R.F. (2001) Visual search has memory. *Psychol. Sci*. **12(4)**, 287–292.
24. Rafal, R.D., Calabresi, P.A., Brennan, C.W., and Sciolto, T.K. (1989) Saccade preparation inhibits reorienting to recently attended locations. *J. Exp. Psychol. Hum. Percept. Perform.* **15**, 673–685.

## FOOTNOTES

[1]The displayed scenes were excerpted from three books by Martin Handford (Grolier Ltd.): *Where's Waldo* (1987) (from which we used: the Beach, the Museum, Department Store [with Waldo], and the Fair); *Find Waldo Now* (1988) (from which we used: Once upon a Saturday Morning [with Waldo] and The Last Days of the Aztecs [with Waldo]; and *The Giant Waldo Search* (1989) (from which we used: The Great Ball Game Players [with Waldo], the Deep Sea Divers). For further information contact: ray.klein@dal.ca

[2]We intended to present probes based on the two-back fixation location, however due to a programming oversight probe positions were based on the one-back location. A serendipitous oversight because the robust IOR obtained in the one-back condition here, after a pause in searching, turned out to be quite informative.

[3]These findings differ in two ways from Klein and MacInnes[1]. First, there was a higher probability of a blink in the present study, which we believe can be attributed to the fact that the present trials were longer than the previous ones primarily because of the longer fixation durations when observers stopped. Second, in the present study participants were more likely to acquire the probe in one saccade (54 vs. 80%). This is a simple consequence of the fact that probes in the previous studies, which were delivered unexpectedly at the beginning of a fixation (randomly selected as far as the participant was concerned), interrupted search, whereas probes in the current study could only occur when the participants had already stopped and were therefore expecting probe presentation.

[4]As in Klein and MacInnes[1], the six different angles were collapsed into four angular distances: 0, ±60, ±120, and 180 (as shown in Fig. 2).

[5]The most obvious explanation for this trend is that with a relatively high probability, observers determine that they had fixated something interesting just after leaving, and hence make a return saccade about 30% of the time in order to stop.

[6]It might be argued that the benefit of this voluntary cessation of search would be offset by the fact that we prompt the subject to continue when probe presentation was not possible (the average frequency of “resume” signals was 1.2 times per trial). If this were encouraging active preparation of the next saccade, which in turn was responsible for the IOR we have observed, then we would expect a correlation between the frequency with which an observer experienced the resume signal and the amount of IOR they showed. Using a measure of IOR that took advantage of the most data contributed by an observer ([0º + 60º] minus [120º + 180º]), we found that there was, in fact, no relation (r = 0.015) between the frequency with which observers had to resume searching after stopping and IOR.

[7]By drawing this conclusion we do not mean to challenge the finding that the previous preparation of a saccade[24] can cause IOR. In that case the inhibition is in the opposite direction to the previous preparation. Here we are interested in whether, when saccades in some directions are faster than others, this is because of current (rather than previous) preparation in the faster direction or because of previously established inhibitory tags in the slower direction.

## BIOSKETCHES:

W. Joe MacInnes is currently in his final year of an Interdisciplinary Ph.D. (Computer Science and Cognitive Psychology) at Dalhousie University. His qualifications include a Bachelor of Computer Science (Sexton Scholar) from the Technical University of Nova Scotia and several years experience as a programmer/researcher for visual and cognitive studies. His research interests include: Human factors, human and computer vision, depth perception, visual search, inhibition of return, user modeling, 3D graphics (Open-GL) and machine learning.

Raymond M. Klein is a Killam Professor of Science in Psychology at Dalhousie University and past president of the Canadian Society for Brain, Behavior and Cognitive Science. He is an experimental psychologist and cognitive neuroscientist whose research strategies are most strongly influenced by those of D. O. Hebb, Donald Broadbent and Michael Posner. His research interests include: Visual attention and its disorders, reading and dyslexia; applied cognitive psychology.
